# Fully (Re)configurable Interactive Material through a Switchable Photothermal Charge Transfer Complex Gated by a Supramolecular Liquid Crystal Elastomer Actuator

**DOI:** 10.1021/jacs.3c05905

**Published:** 2023-08-23

**Authors:** Shuang Tian, Sean J. D. Lugger, Chun-Sing Lee, Michael G. Debije, Albert P. H. J. Schenning

**Affiliations:** † Center of Super-Diamond and Advanced Films (COSDAF) and Department of Chemistry, City University of Hong Kong, Hong Kong SAR 999077, P. R. China; ‡ Stimuli-Responsive Functional Materials and Devices (SFD), Department of Chemical Engineering and Chemistry, Eindhoven University of Technology, P.O. Box 513, 5600 MB Eindhoven, The Netherlands; § Institute for Complex Molecular Systems (ICMS), Eindhoven University of Technology (TU/e), Groene Loper 3, 5612 AE Eindhoven, The Netherlands; ∥ Interactive Polymer Materials (IPM), Eindhoven University of Technology (TU/e), Groene Loper 3, 5612 AE Eindhoven, The Netherlands

## Abstract

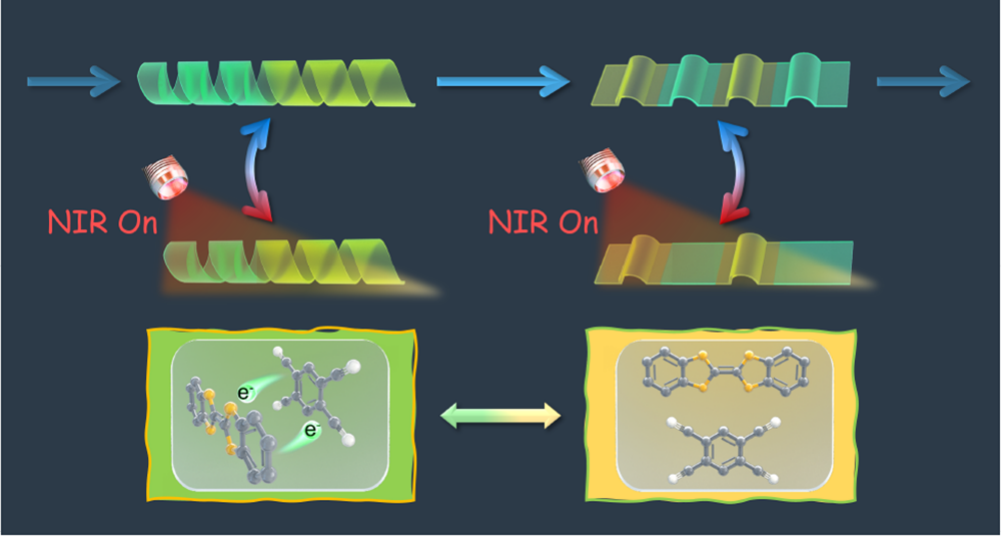

Charge transfer complexes (CTCs) based on self-assembled donor and acceptor molecules allow light absorption of significantly redshifted wavelengths to either the donor or acceptor. In this work, we demonstrate a CTC embedded in a hydrogen-bonded liquid crystal elastomer (LCE), which in itself is fully reformable and reprocessable. The LCE host acts as a gate, directing the self-assembly of the CTC. When hydrogen bonding is present, the CTC behaves as a near-infrared (NIR) dye allowing photothermal actuation of the LCE. The CTC can be disassembled in specific regions of the LCE film by disrupting the hydrogen bond interactions, allowing selective NIR heating and localized actuation of the films. The metastable non-CTC state may persist for weeks or can be recovered on demand by heat treatment. Besides the CTC variability, the capability of completely reforming the shape, color, and actuation mode of the LCE provides an interactive material with unprecedented application versatility.

## 1 Introduction

Stimuli-responsive materials have attracted much attention for applications ranging from soft robots and actuators to optical sensors.^1−4^ Responsive materials change their functional properties, including shape and/or color, when exposed to a stimulus, and, upon stimulus removal, return to their initial stable state.^5,6^ Interactive materials are regarded as the next step from stimuli-responsive materials to materials that adapt and respond to internal and external stimuli in a coupled manner.^7^ Two common approaches for fabricating responsive materials are the self-assembly of molecules and supramolecular polymers.^8−12^ In this work, we merge these two fields and use a supramolecular liquid crystal (LC) polymer actuator to control the assembly and disassembly of a near-infrared (NIR) photothermal charge transfer complex (CTC). This regulation results in a NIR light-driven interactive material that has (re)programmable, intricate geometrical (meta)stable shapes with multiple actuation modes.

CTCs based on non-covalent, self-assembled donor (D) and acceptor (A) molecules have been used for engineering optical materials.^13−17^ Energy gaps of CTCs can be tuned by adjusting interactions between their constituent donors and acceptors, leading to controllable optical absorption with significantly redshifted wavelengths relative to the individual components.^18−20^ Non-covalent interactions are reversible and sensitive to environmental factors, including temperature and solvent, making CTCs attractive for creating responsive systems, although such systems are rarely reported.^21,22^ Light, thermal, and electrical stimuli have been used to fabricate multistate CTC-based thin-film ferroelectric memory devices able to reversibly bend by desorption and absorption of solvent.^23^ The use of CTCs in stimuli-responsive polymer materials has not yet been reported.

Liquid crystal elastomers (LCEs) are able to respond to environmental triggers, including temperature and light, resulting in rapid, reversible, and complex motions.^24−29^ Light-responsive actuators are particularly interesting as they allow untethered actuation from a distance.^30,31^ Making LCE films responsive to light is often done by the addition of a photothermal material that converts light into heat, disrupting local order and causing actuation.^32−35^ Light-driven soft actuators with programmable, multiple arbitrary stable rest states or actuation modes have been reported.^31,36−38^ However, it remains challenging to fabricate light-driven, versatile soft actuators with fully reprogrammable, pre-designed rest states and shape deformations from the same LCE film.

Recently, we reported melt-processable supramolecular LCE actuators based on segmented copolymers containing hydrogen-bonded (H-bonded) thiourethane “hard” and LC “soft” segments.^39^ By exploiting the supramolecular H-bonded cross-links, the polymer could be molded, recycled, and reprogrammed.

In this work, we generate a switchable CTC within a responsive H-bonded LCE, moving toward an interactive NIR-responsive material. The LCE host acts as a gate, directing self-assembly of the CTC when H-bonding is present, resulting in a NIR absorption band allowing photothermal actuation of the LCE. Upon breaking the H-bonds in the LCE, the individual D/A molecules separate, creating a metastable film that no longer actuates since neither the acceptor nor donor absorbs NIR light. The H-bonded LCE itself can be completely reconfigured and reformed with both different shapes and locally programmed actuation modes with minimal difficulty and waste of material, a step toward meeting the stringent requirements demanded for sustainable interactive materials.

## 2 Results and Discussion

The energy gap of a CTC is typically defined by the energy offset between the highest occupied molecular orbital (HOMO) of the donor and lowest unoccupied molecular orbital (LUMO) of the acceptor. In the present system, the yellow-colored DBTTF (donor; calc. HOMO = −4.7 eV) and the white TCNB (acceptor; calc. LUMO = −3.9 eV) form a CTC with an energy gap of 1.3 eV, corresponding to an absorption edge of ∼903 nm (Figures S1–S3). This matches well with density functional theory (DFT) calculations (Figure S4).^18^ Our earlier-reported supramolecular polymer containing thermally dynamic H-bonds was selected as the LCE host.^39,40^ The supramolecular LCE provides well-defined, microphase-separated LC soft domains, alignment conducive to actuation, and dynamic H-bonded hard segments, allowing reforming and reprogramming.

**Figure 1:**
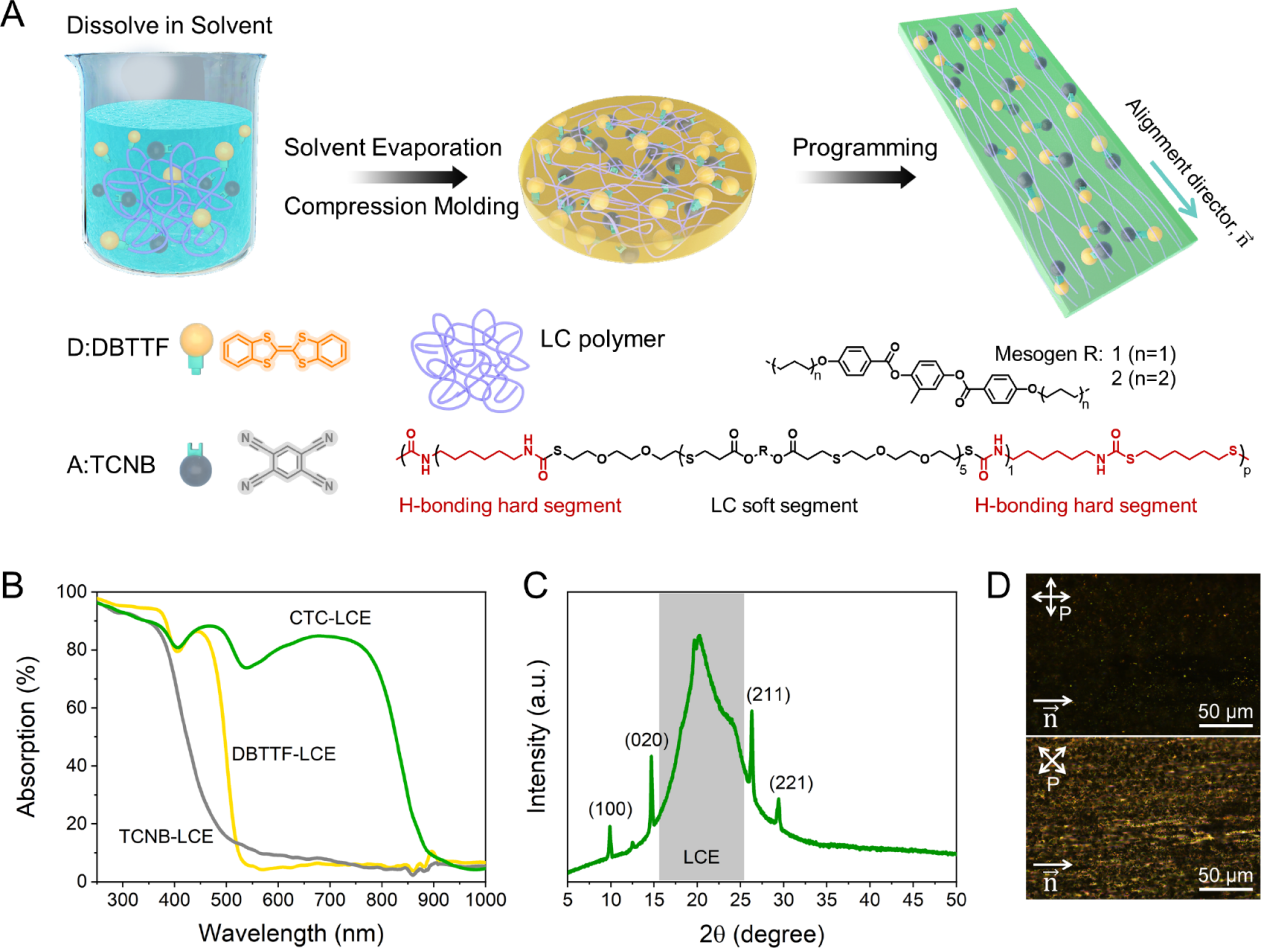
(A) Molecular representation and schematic illustration of the CTC-LCE actuator preparation. (B) Normalized absorption spectra of the donor DBTTF-LCE (1.26 wt %), acceptor TCNB-LCE (0.74 wt %), and CTC-LCE (2 wt %) in the LCE host. (C) XRD spectra and (D) POM images with cross polarizers of the CTC-LCE actuator (2 wt %). The alignment director is indicated as “*n⃗*”.

To prepare the CTC-LCE actuators (Figure 1A), the LCE and equimolar DBTTF donors and TCNB acceptors are dissolved in an organic solvent mixture (chloroform:1,1,3,3,3-hexafluoro-2-propanol = 6:1). Upon drying, the resulting material is compression-molded into homogeneous ∼0.3 mm-thick films and stretched at 130 °C to 100% strain to align the LCE. CTC formation as a function of the doping ratio in the LCE is immediately visualized by the obvious color differences (Figures S5 and S6). The green color and the absorption spectrum of the CTC-LCE actuator with a 2 wt % doping ratio (Figure 1B, absorption peak at 682 nm; individual absorption peaks for donor DBTTF and acceptor TCNB in LCE are 444 and 308 nm, respectively) are very close to the color and absorption of the CTC cocrystals and are used for all subsequent experiments (Figures S2 and S3).

The X-ray diffraction (XRD) pattern of this 2 wt % CTC-LCE film reveals diffraction peaks corresponding to the CTC and LCE (Figure 1C and Figures S7 and S8).^39,41,42^ Molecular alignment of the CTC-LCE is confirmed by polarized optical microscopy (POM) and X-ray (Figure S9): the stretched LCE exhibits birefringence upon rotating the crossed polarizers with an initial X-ray determined order parameter of ∼0.27, while the CTC microcrystals are randomly orientated (Figure 1D and Figure S10). These results reveal the successful fabrication of a self-assembled CTC in an anisotropic supramolecular LC polymer film.

**Figure 2:**
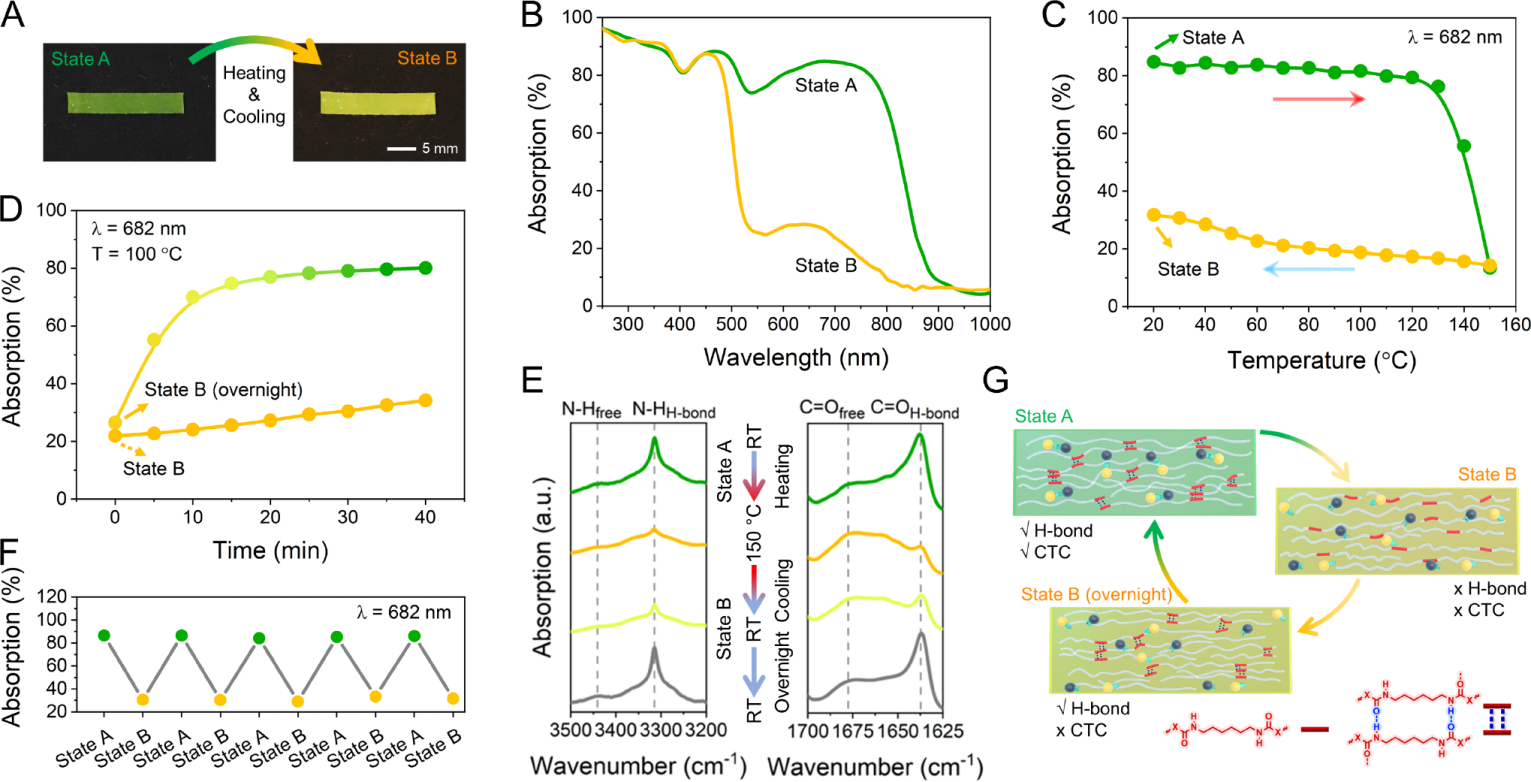
(A) Images of the 2 wt % CTC-LCE actuator in different states and the transition scheme. (B) Normalized absorption spectra and (C) temperature-dependent normalized peak absorption (λ_max_ = 682 nm) of the CTC-LCE actuator in States A and B. (D) Time-dependent normalized absorption of the CTC-LCE actuator in State B at 100 °C immediately after thermal treatment (heating to 150 °C and cooling to RT) or after storing overnight at RT (yellow and yellow-green line, respectively). (E) FTIR spectra of amine (left, 3200–3500 cm^–1^) and carbonyl vibrations (right, 1625–1700 cm^–1^) through one entire heating/cooling cycle and storing overnight. (F) Peak absorption of five transition cycles (heated to 150 °C for 10 min, cooled to RT, and spectra immediately recorded in State B (yellow dots), followed by overnight storage at RT to recover H-bonds, and followed by heating to 100 °C for 40 min to recover State A (green dots)). (G) Schematic illustration of the postulated situation of the 2 wt % CTC-LCE actuator in different states.

The thermochromic behavior of the polymer film was investigated by recording temperature-dependent absorption spectra (Figure 2A,B) and monitoring the CTC absorption peak changes at 682 nm during both heating and cooling at 10 min intervals (Figure 2C and Figures S11 and S12). The absorbance remains unchanged upon heating from 20 to 120 °C, indicating that the CTC remains intact. When increased above 120 °C, the CTC absorption decreases sharply, and the film’s color changes from green to yellow, suggesting that acceptors and donors separate (vide infra): optical microscopy indicates that CTC crystals disappear upon heating and do not grow in size (Figure S13). Surprisingly, cooling from 150 to 20 °C follows a different trajectory as the yellow color persists (Figure 2C). Storing the CTC-LCE at room temperature (RT) overnight does not immediately return the system to green-colored State A: it instead forms a metastable, yellow-colored State B with maintained alignment (Figure S9). Full recovery of the green color at RT took around 2 weeks (Figure S14). To investigate the stability of the yellow-colored State B, recovery to the green-colored State A was measured at different temperatures. When the yellow State B film, which has been stored at RT overnight, is heated to an LCE isotropic temperature of 100 °C, the green color appears again within 40 min (yellow-green line in Figure 2D). The CTC-LCE showed fully reversible switching between States A and B over at least five transition cycles (Figure 2F). Remarkably, however, when the yellow-colored State B film is immediately heated to 100 °C (i.e., without the overnight storage), the yellow color remains (yellow line in Figure 2D). Immediately cooled to and maintained at 4 °C, the CTC-LCE returns to State A in 30 days (Figure S15). If the sample is instead cooled to and maintained at −18 °C, then State B persists for more than 1 month (Figure S16). This data indicates that the film switches from an initial green CTC State A (>85% absorbance at 682 nm) into a metastable yellow non-CTC State B (∼23% absorbance at 682 nm) by thermal treatment at 150 °C and storing at RT (Figure 2A,B). To confirm this hypothesis, XRD was performed, revealing that, indeed, the yellow-colored film (Figure S17) shows a near-complete absence of CTC peaks at RT.

To investigate the origin of the formation of State B, the H-bonding in the LCE as a function of temperature was monitored by following the amine and carbonyl signals using Fourier-transform infrared (FTIR) spectroscopy (Figure 2E). At RT, the N–H_H-bond_ stretching band appears as a sharp vibration (3315 cm^–1^), while the N–H_free_ is only weakly observed, mirrored by sharp C=O_H-bond_ (1638 cm^–1^) and minor free C=O (1677 cm^–1^) stretching bands. These spectra suggest H-bonding between N–H_H-bond_ and C=O_H-bond_ in the LCE. During heating, these signals display little changes up to 120 °C. A disruption of the H-bonding network measured by decreases in the corresponding FTIR vibrations was observed from 130 to 150 °C (Figure S18). Interestingly, H-bonding is only fully recovered after cooling and storing at RT overnight (Figures S19 and S20): the melting point of the DBTTF-TCNB cocrystal itself is around 330 °C.^18^ These results correspond with the disassembly of the CTC and the delayed reassembly at RT, as discussed above.

Based on these observations, we postulate a segmented environment in the LCE where the CTC is located in the LC soft segment. Upon breaking the H-bonds in the hard segments at higher temperatures, a single phase is created, and the CTCs dissociate into acceptor and donor components yielding the metastable, yellow-colored State B (Figure 2G). The phase change upon reforming the H-bond network enforces the eventual reassembly of the CTC in the LC segments restoring the green color (State A). We support this argument with experiments comparing the 682 nm peak absorption and C=O_H-bond_/C=O_free_ bond ratios as a function of temperature in the A state (Figure S21) and in the B state as functions of temperature and time (Figure S22).

Interestingly, the LCE host appears to act as a gate, directing the self-assembly of the CTC. Maintaining a just-formed State B sample at RT overnight allows the reforming of the H-bonds, but the sample remains yellow, meaning that the CTC has not yet reformed. When the H-bonds are more completely formed after sitting overnight, the space for the donor and acceptor is now constricted, but their mobility in the LCE is quite limited, so CTC reformation is very slow (days to weeks). When heated to 100 °C for 30 min, the H-bonds partially dissociate, but the integrity of the structure is mostly maintained; the mobility of the D/A significantly increases, and there is a dramatically increased probability of D/A encounter and reforming of the CTCs (and recovery of the green color).

**Figure 3:**
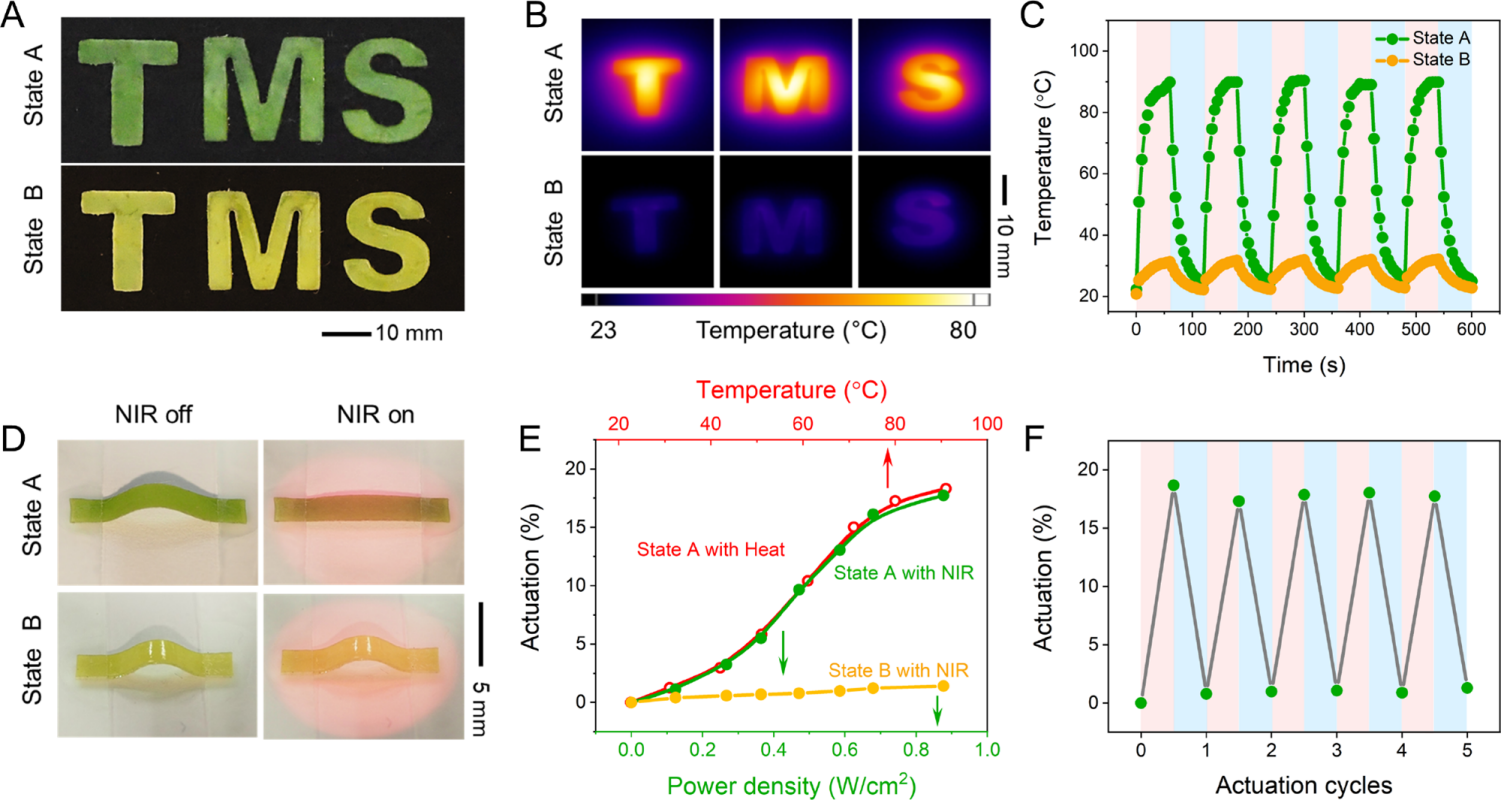
(A) Photographs of the CTC-LCE film in different states. (B) Photothermal images of the CTC-LCE film in different states under 0.7 W/cm^2^ illumination. (C) Temperature evolution of the CTC-LCE in different states during five heating/cooling cycles under 0.9 W/cm^2^ illumination. (D) Photographs of buckled CTC-LCE films in States A and B under photothermal actuation with 0.7 W/cm^2^ illumination. (E) Thermal actuation curve of the CTC-LCE in State A and photothermal actuation curves of the CTC-LCE in States A and B under LED illumination with different power densities. (F) Reversible photothermal actuation generated during five LED illumination cycles. A 780 nm LED source is used for photoactuation.

Inspired by the optical differences between States A and B, CTC-LCE sheets were cut into shapes of the letters T, M, and S (Figure 3A), and their photothermal responses were investigated. The green and yellow (obtained after heating to 150 °C and cooling to RT) colored letters were irradiated with a 780 nm NIR light-emitting diode (LED) for 60 s; surface temperature of the green color letters reached 77 °C, while yellow-colored letters reached only 28 °C (Figure 3B). Clearly, NIR is absorbed, and heat is generated in the film only when the CTC is present in the polymer sheets. Both states attained steady-state temperatures after ∼60 s of irradiation (Figure S23). Illumination at higher intensities led to higher steady-state temperatures (Figure S24). Repeating the heating and cooling cycles five times on a thicker sample using 0.9 W/cm^2^ irradiation for 60 s each demonstrated that the State A CTC-LCE film reproduces the same temperature rise to 91 °C, while State B does not heat beyond 31 °C (Figure 3C). These results reveal that the CTC embedded in the LCE film might be used as a switchable NIR photothermal dye.

We studied the NIR photothermal actuation of aligned CTC-LCE films (Figure 3D). Upon exposure to NIR light, the green-colored polymer film contracts, and by increasing the power density of the light, a maximum contraction of 19% is obtained (Figure 3E). The film exhibits fully reversible actuation over at least five irradiation cycles, demonstrating the photothermal stability of the actuator (Figure 3F). Interestingly, the NIR photothermal actuation curve overlaps with the pure thermal actuation curve recorded over a range of 20–90 °C (Figure 3E), demonstrating a maximum contraction of 19%, similar to LCE samples without the CTC.^39,43^ In contrast, there is no significant NIR photothermal actuation of the film in the yellow-colored State B films (Figure 3D). However, direct thermal exposure gives similar deformation as the green-colored film (Figure 3E).

**Figure 4:**
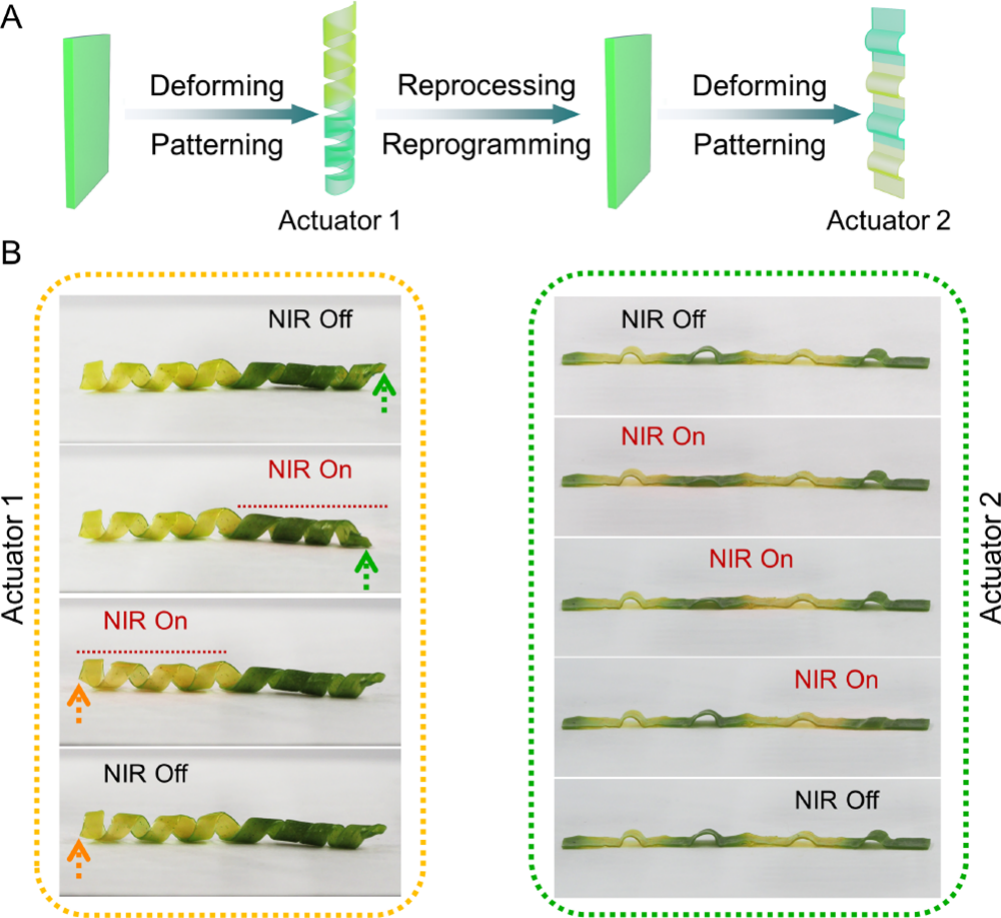
(A) Schematic preparation of 3D actuators using a single CTC-LCE film with different shapes in the rest state (actuator 1 and 2) and actuation modes. (B) Photographs of the NIR selective actuation for the reconfigurable CTC-LCE actuator.

To demonstrate the potential of this novel CTC-LCE system as a versatile soft actuator with programmable multiple rest states and actuation modes, two actuators were prepared sequentially from the same polymer film. The initial actuator was formed by stretching and wrapping a film around a cylindrical template at 130 °C to form an extended coil. The coil was regionally heated to 180 °C in a mold over one-half its length and then allowed to cool to RT, forming a patterned, coil shape actuator with yellow and green colors on opposite ends (Figure 4A). This actuator was irradiated with a 780 nm LED from above. Irradiation on the yellow region generated no actuation as expected, while the green side responded by absorbing the incident radiation, causing the LCE to heat up and generating disorder of the chains, resulting in contraction and uncoiling into a looser coil (from 4.5 to ∼4 turns; Figure 4B left and Video S1).

**Figure 5:**
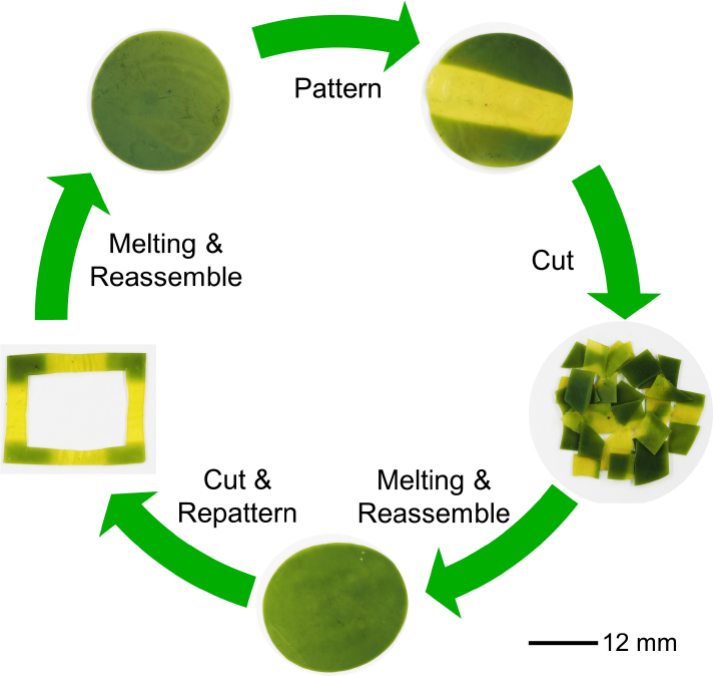
Photographs of the entire reprocessing and repatterning cycle of the CTC-LCE.

The coil was then reprogrammed by raising the temperature to 180 °C, breaking all the H-bonds and dissociating the CTC into acceptors and donors. Afterward, the pliable material was stretched and embossed by a mold at 130 °C to form a strip with periodic “hill” projections (Figure 4A). Specific regions were heated in the mold to 180 °C and left to cool at RT to form yellow regions within the green film. Light (780 nm) was used to illuminate the patterned sample: while the yellow regions remained unaffected, the green regions actuated by flattening and then returning to their “bumpy” form after the removal of the light source (Figure 4B right and Video S2). These results reveal that it is possible to fabricate light-driven soft actuators with pre-designed arbitrary rest states and shape deformations from the same reusable LCE film. By using higher-power light sources, it is also possible to generate local temperatures around 150 °C and local patterning of larger areas with high precision to allow more complex responses (see Figure S25). However, stepping even beyond the CTC variability, the CTC-LCE itself can be reprocessed in its entirety. After chopping the material into pieces, they may be reformed into any desired structure, which may then be addressed by light to create the responsive green and non-responsive yellow regions (Figure 5).

## 3 Conclusions

A self-assembled D/A system embedded in a H-bonded LCE host may be triggered to reversibly form CTCs capable of absorbing NIR light, which is not absorbed by the individual component molecules. This generates local heating and actuation of the host polymer when exposed to NIR light. The films can be locally addressed by temperature to disassemble these complexes, rendering specific regions incapable of actuation upon NIR light exposure. The initial CTC state may be recovered in less than 1 h and is gated by the LCE *via* H-bond interactions. The sequestration of a single film into locally actuating and non-actuating regions can be done by patterned light exposure and results in fully (re)configurable soft actuators.

The (re)configuring of materials into different forms and performing new functions could help reduce the generation of waste that becomes space junk.^44−47^ As another example, consider CTC-LCEs acting first as soft actuators and later repurposed as reusable optical sensors (Figures S15 and S16).^48,49^ Our results show that merging the self-assembly of molecules and supramolecular polymers results in a new class of interactive, sustainable materials with coupled and gated responses.

## Supporting Information Available

Photothermal actuation of the patterned coiled actuator (MP4)
Photothermal actuation of the patterned strip with periodic “hill” projections (MP4)
Preparation methods, experimental section, and additional characterization details (PDF)
