# Supplementary material for: Fully (Re)configurable Interactive Material through a Switchable Photothermal Charge Transfer Complex Gated by a Supramolecular Liquid Crystal Elastomer Actuator
Source: J Am Chem Soc. 2023 Aug 23;145(35):19347–53. doi: 10.1021/jacs.3c05905 (PMC10485926; doi:10.1021/jacs.3c05905)
Supplement: Supplementary file 3 — ja3c05905_si_003.pdf [file ja3c05905_si_003.pdf]

Supporting Information

Fully (Re)configurable Interactive Material through a Switchable Photothermal Charge Transfer Complex Gated by a Supramolecular Liquid Crystal Elastomer Actuator

Shuang Tian[1,2,†], *Sean J. D. Lugger*[2,3,†], *Chun-Sing Lee**[1], *Michael G. Debije**[2,3,4] *and Albert P. H. J. Schenning**[2,3,4]

[1] Center of Super-Diamond and Advanced Films (COSDAF) and Department of Chemistry, City University of Hong Kong, Hong Kong SAR, 999077 P. R. China

[2] Stimuli-responsive Functional Materials and Devices (SFD), Department of Chemical Engineering and Chemistry, Eindhoven University of Technology, P.O. Box 513, 5600 MB Eindhoven, The Netherlands.

[3] Institute for Complex Molecular Systems (ICMS), Eindhoven University of Technology (TU/e), Groene Loper 3, 5612 AE Eindhoven, The Netherlands

[4] Interactive Polymer Materials (IPM), Eindhoven University of Technology (TU/e), Groene Loper 3, 5612 AE Eindhoven, The Netherlands

[†]These authors contributed equally: Shuang Tian, Sean J. D. Lugger.

[*]Address correspondence to: E-mail: apcslee@cityu.edu.hk, m.g.debije@tue.nl, a.p.h.j.schenning@tue.nl.

EXPERIMENTAL SECTION

Materials

Dibenzotetrathiafulvalene (DBTTF) was obtained from Sigma-Aldrich. 1,2,4,5-tetracyanobenzene (TCNB) was purchased from J&K Scientific Ltd. Acetonitrile (ACN), methanol (MeOH), chloroform ($CHCl_3$), and 1,1,3,3,3-hexafluoro-2-propanol (HFIP) were obtained from Biosolve. Unless otherwise stated, all chemicals were used without further purification. The hydrogen-bonded LCE host was synthesized based on our previous work.[1,2] To prevent crystallinity and the formation of a smectic mesophase in the LC soft segment, an equimolar mixture of two mesogens (n = 1 or 2) was used.

Characterization

Crystal structures were measured with a powder X-ray diffractometer (P-XRD, Bruker D2 PHASER with LYNXEYE XE-T detector) using a CuKα source. Wide-angle X-ray spectroscopy measurements were performed on a Ganesha lab instrument equipped with a Pilatus 300 K silicon pixel detector (487 × 619 pixels, 172 μm^2 in size) and a Genix-Cu ultralow divergence source (CuKα, $\lambda = 0.154$ nm, $\Phi = 1 \times 10^8$ photons s^{-1}). Silver behenate was used as the calibration standard, and the orientational order parameter *S* was determined using the Kratky method.[1,2] Polarized optical microscopy (POM) was carried out with a Leica DM2700 M microscope equipped with crossed polarizers. Reflection spectra were recorded with a UV-Vis-NIR spectrophotometer (Shimadzu 1700) and converted to normalized absorption spectra (%) by using the following equation: absorption (%) = 100 (%) - reflectance (%). Fourier-Transform infrared spectra (FTIR) were collected with a Varian 670 spectrophotometer equipped with attenuated total reflectance accessories. The temperature changes of the actuator were controlled by a Linkam THMS600 hot stage. Thermal images were captured using an infrared camera (Fluke TI32). Photographs and videos were taken with a digital camera (Olympus OM-D E-M10 Mark III). Near-infrared (NIR) light illumination was performed using a 780 nm LED (Thorlabs M780LP1) and LED driver (Thorlabs DC2200).

Theoretical Calculations

Intermolecular potentials were calculated using the UNI force field by Mercury software (copyright CCDC). Calculated BFGH morphology was shown by Mercury software according to the crystal structure. Calculations of the energy levels were made using density functional theory (DFT) by Gaussian 09 package.[3] The crystal structure of the donor-acceptor molecular pair was directly imported, and the molecular orbitals were obtained at the B3LYP/6-31 G* level. The degree of charge transfer was evaluated from Mulliken population analysis.

Preparation of CTC cocrystal

In a typical synthesis, the growth of CTCs was accomplished by mixing equimolar donor (DBTTF) and acceptor (TCNB) in hot ACN. Dark green CTC precipitated by slowly evaporating the solvent. Afterward, the precipitate was washed thrice with MeOH and dried at 80 °C overnight (yielding ~ 95%).

Preparation of flat CTC-LCE actuators

In a typical preparation, the donor (DBTTF) and acceptor (TCNB) were dissolved in equimolar quantities in HFIP and $CHCl_3$ in a 1:6 volume ratio yielding a CT pair solution with 1 mg/mL concentration. The LCE host was added to the CT pair solution in different weight ratios (CTC:LCE = 0:100, 0.25:100, 0.5:100, 1:100, 2:100, and 3:100). Each mixture was stirred vigorously, poured into a Teflon mold, and placed under vacuum at room temperature (RT) overnight to completely evaporate the solvent. CTC-LCE films were obtained by removing the material from the Teflon molds. In the compression-molding process, the CTC-LCE film was compressed three times at 180 °C with 0.5 bar for 2 minutes each. The molded CTC-LCE films were kept at RT for at least 24 hours. Actuators were obtained by stretching the film to 100% elongation and subsequently heating it to 100 °C for 30 minutes. The final actuator was cut from the resultant sheet with a razor blade.

Preparation of 3D shaped CTC-LCE actuator

The coiled actuator was obtained by stretching a CTC-LCE film (30 × 3 mm^2) to 100% elongation, wrapping it around a cylindrical support at 130 °C for 30 minutes, and cooling it to RT for 30 minutes. The coil was then selectively heated in the mold to 180 °C for 1 minute over one-half of its length and left at RT for 30 minutes, after which the mold was removed, yielding a patterned coil with State B on one end and State A on the other end. After actuation with NIR light, the coil was reprocessed back into the initial CTC-LCE film by heating it to 180 °C for 2 minutes, erasing the shape and pattern, and left at RT overnight. Next, a strip with periodic 'hill' projections was obtained by stretching (100%) and embossing the film (30 × 5 mm^2) in a mold at 130 °C for 30 minutes and subsequently cooling to RT for 30 minutes. Specific regions of the film were then selectively heated in the mold to 180 °C for 1 minute and left to cool at RT for 30 minutes, after which the mold was removed, yielding a patterned film with alternating state A and state B 'hill' projections.

Reprocessing of the patterned CTC-LCE film

A pristine CTC-LCE film was patterned by local heating to 180 °C for 1 minute and then cooled to RT. The patterned film is then cut into small pieces. All small pieces are remolded at 180 °C with 0.5 bar for 2 minutes, stored overnight at RT, and heated at 100 °C for 40 minutes to obtain the original film before reprocessing. Next, a 'frame' shaped CTC-LCE was cut from the pristine film and selectively patterned by locally heating specific regions to 180 °C for 1 minute and cooling it to RT. Finally, the

‘frame’ shaped CTC-LCE and the remainder of the original pristine film were recovered by repeating the reprocessing step again (**Figure 5** in the main text).

Thermal actuation measurements

The CTC-LCE actuator was placed on a hot plate and gradually heated from RT (22.4 °C) to 90 °C with intervals of 10 °C. Afterward, the samples were allowed to cool to RT. All samples were subjected to a full heating and cooling cycle to erase the thermal history before all actuation measurements. Photographs were taken at each temperature using a digital camera, and the resulting images were analyzed using the open-source software ImageJ.

Photothermal actuation measurements

CTC-LCE actuators were placed under NIR light (780 nm LED) and stepwise irradiated from 0 to 0.9 W/cm^2. Afterward, the samples were allowed to cool to RT. All samples were subjected to a full heating and cooling cycle to erase the thermal history before all actuation measurements. Photographs were taken at each temperature using a digital camera, and the resulting images were analyzed using the open-source software ImageJ.

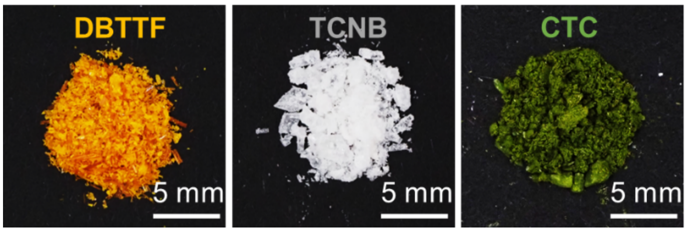

Figure S1. Photographs of DBTTF, TCNB, and CTC samples (left to right).

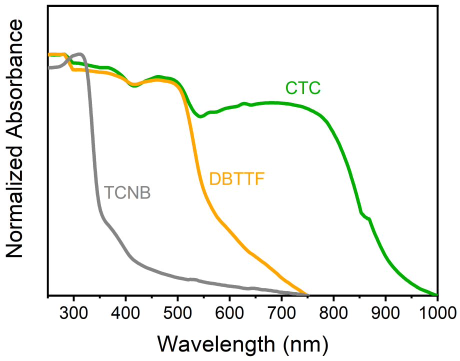

Figure S2. Normalized absorption spectra of TCNB, DBTTF, and CTC samples.

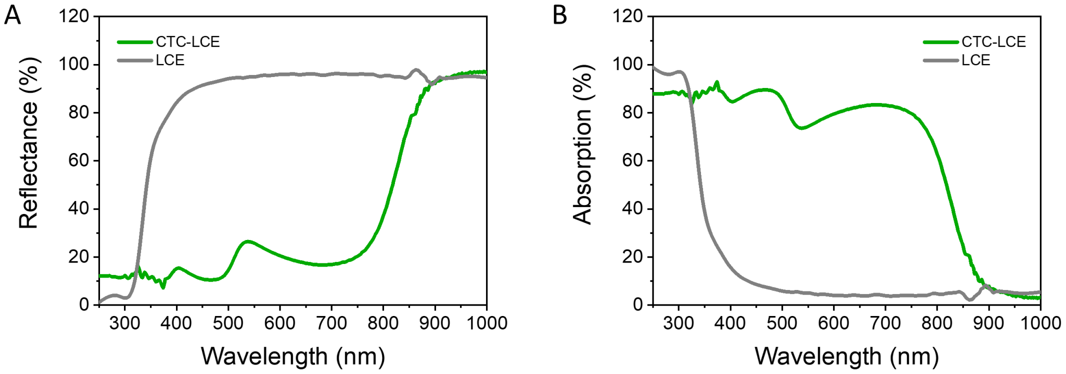

Figure S3. a) Measured reflection spectra and b) corresponding normalized absorption spectra of the CTC-LCE (2 wt%) and LCE actuators.

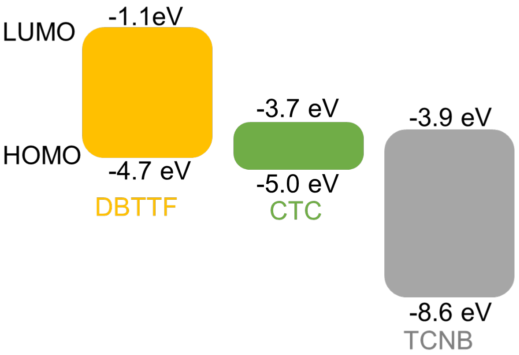

Figure S4. DFT calculated energy diagrams of DBTTF, CTC, and TCNB.

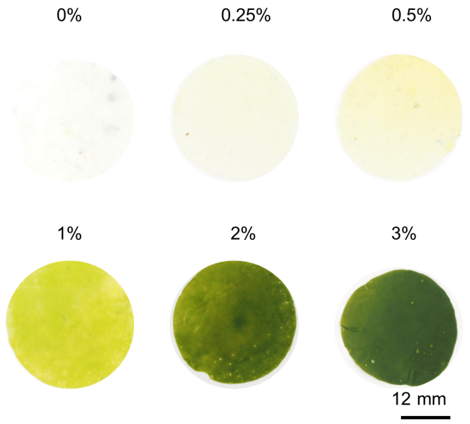

Figure S5. Photographs of CTC-LCE films with different CTC doping ratios.

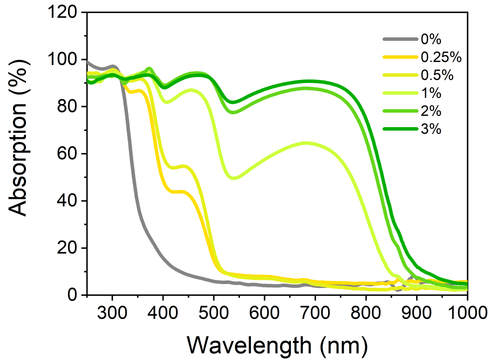

Figure S6. Normalized absorption spectra of the CTC-LCE with different CTC doping ratios.

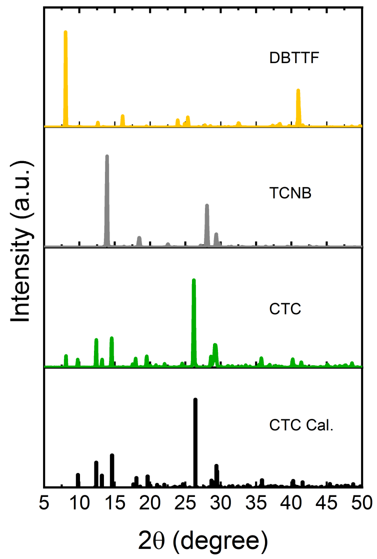

Figure S7. XRD spectra of DBTTF, TCNB, CTC, and the calculated CTC spectrum.

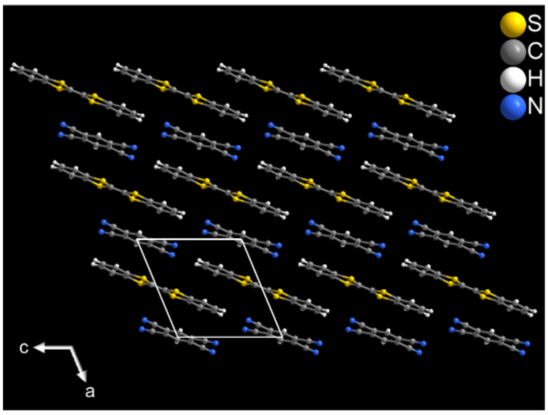

Figure S8. Crystal structure of the CTC.[4]

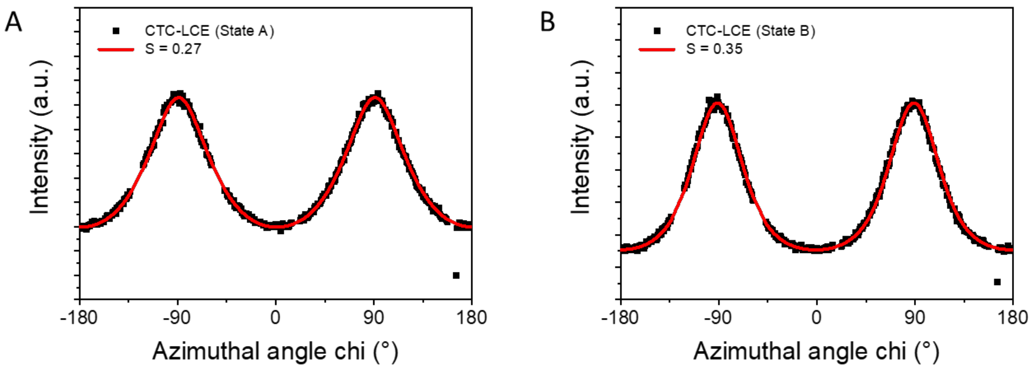

Figure S9. Azimuthal profile of the 2D WAXS diffractogram at scattering vector q = 14.3–14.4 nm^{-1} for the CTC-LCE in A) State A and B) State B (heated to 150 °C for 10 min while the length is fixed).

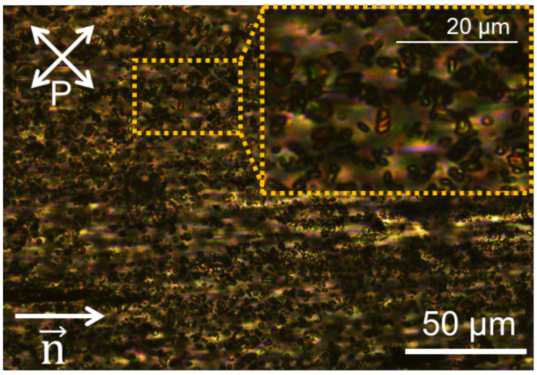

Figure S10. POM image with cross polarizers of the CTC-LCE actuator (2 wt%): inset is a zoomed-in image displaying the random orientation of embedded CTC crystals.

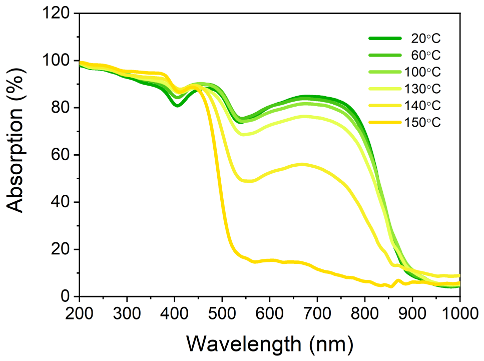

Figure S11. Normalized temperature-dependent absorption spectra of the CTC-LCE actuator upon heating.

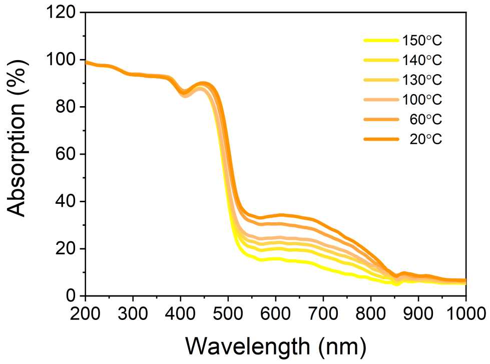

Figure S12. Normalized temperature-dependent absorption spectra of the CTC-LCE actuator upon cooling.

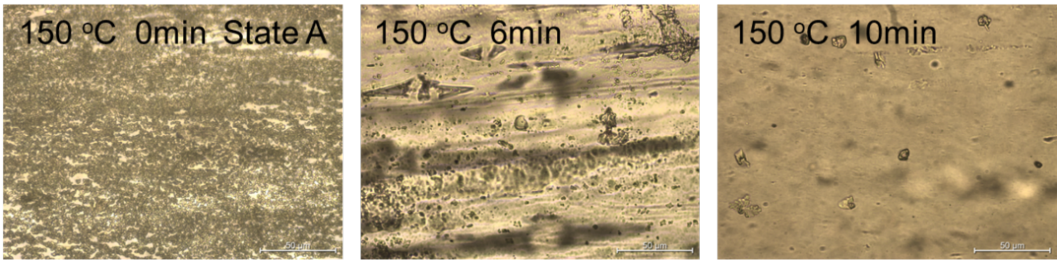

Figure S13. Optical microscope images of the CTC-LCE after heat treatment for the indicated time periods.

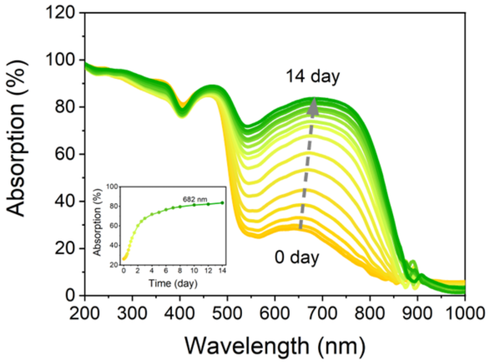

Figure S14. Normalized absorption spectra of the CTC recovery in the LCE film at RT.

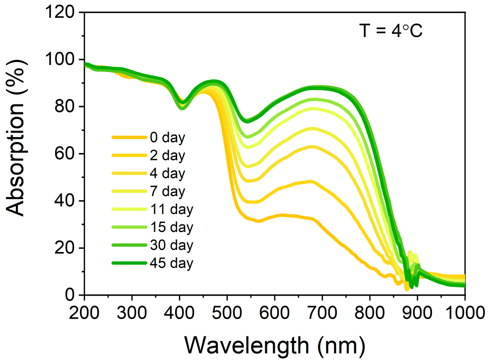

Figure S15. Normalized absorption spectra of the CTC recovery in the LCE film at 4 °C.

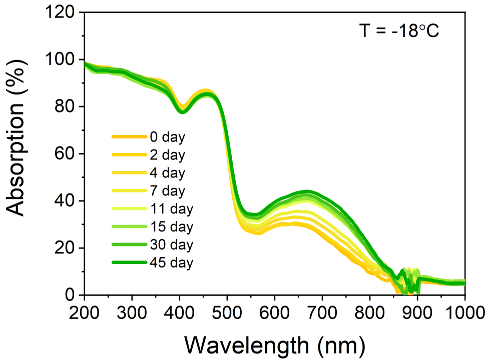

Figure S16. Normalized absorption spectra of the CTC recovery in the LCE film at -18 °C.

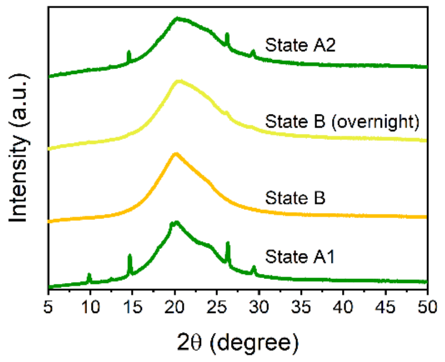

Figure S17. XRD spectra of the different transition states in the CTC-LCE film: The initial State A1 at RT, heated to 150 °C and cooled back to RT (State B), stored overnight at RT (State B (overnight)), and heated to 100 °C for 40 minutes and cooled back to RT (State A2).

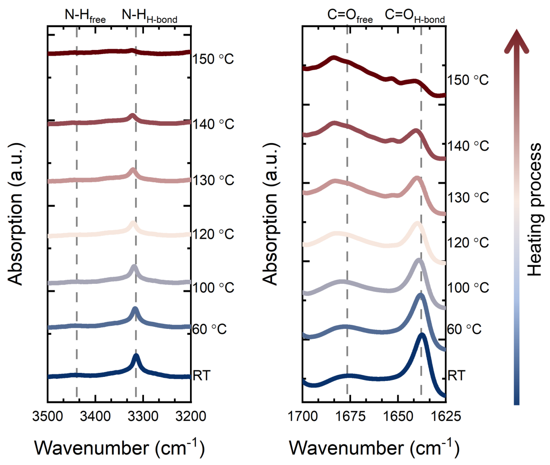

Figure S18. FTIR spectra of the CTC-LCE film upon heating.

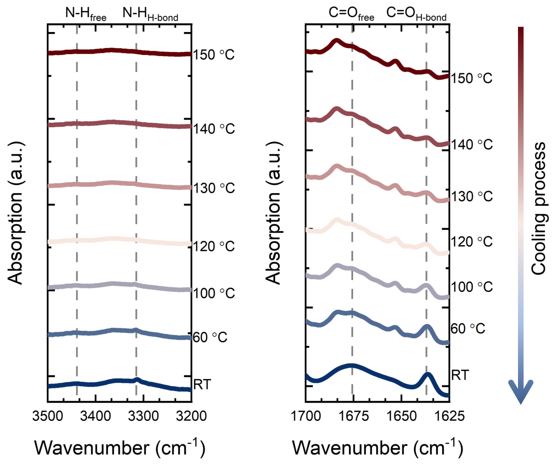

Figure S19. FTIR spectra of the CTC-LCE film upon cooling.

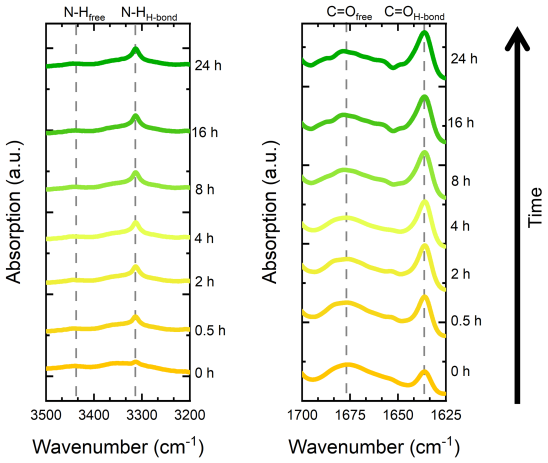

Figure S20. Time-dependent FTIR spectra of the CTC-LCE in State B at RT.

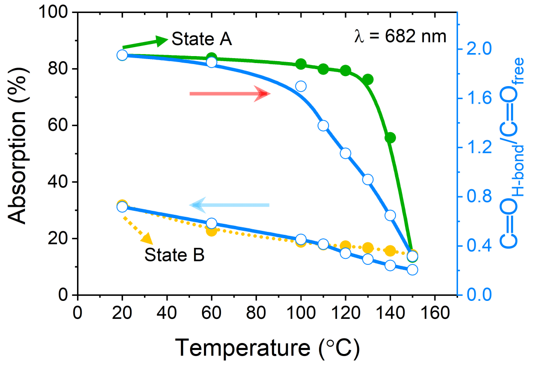

Figure S21. Temperature-dependent peak absorption (λ_{max} = 682 nm) and FTIR C=O$_{H\text{-}bond}$/C=O$_{free}$ ratio changes of the CTC-LCE actuator upon heating and cooling.

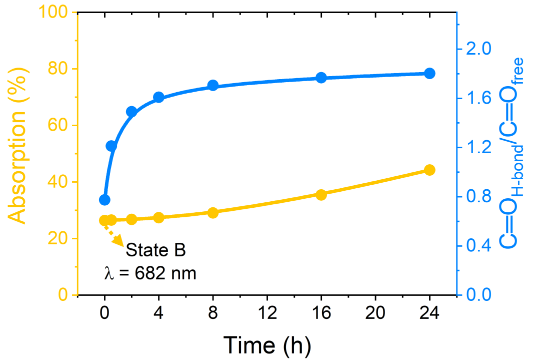

Figure S22. Time-dependent peak absorption (λ_{max} = 682 nm) and FTIR C=O$_{H\text{-}bond}$/C=O$_{free}$ ratio changes of the CTC-LCE actuator in State B at RT.

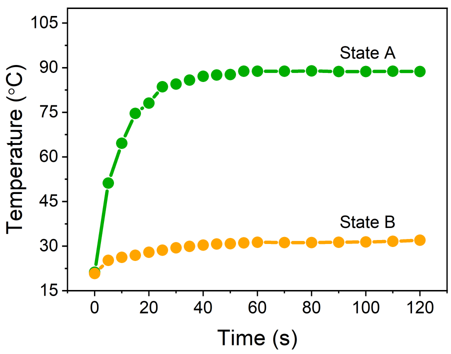

Figure S23. Temperature increase of the CTC-LCE in the different states upon exposure to NIR light (780 nm, 0.9 W/cm^2).

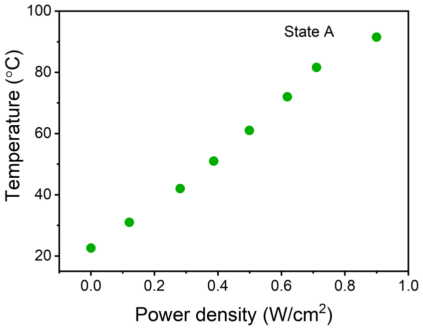

Figure S24. Photothermal effect of the CTC-LCE as a function of incident power density (780 nm).

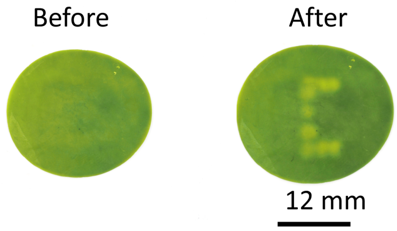

Figure S25. Images of NIR light writing (780 nm, 2W/cm^2, 60 s light exposure for each dot).

References

[1] S. J. D. Lugger, D. J. Mulder, A. P. H. J. Schenning, One-Pot Synthesis of Melt-Processable Supramolecular Soft Actuators, *Angew. Chem. Int. Ed.* **2022**, *61*, e202115166.

[2] S. J. D. Lugger, L. Ceamanos, D. J. Mulder, C. Sánchez-Somolinos, A. P. H. J. Schenning, 4D Printing of Supramolecular Liquid Crystal Elastomer Actuators Fueled by Light, *Adv. Mater. Technol.* **2023**, *8*, 2201472.

[3] Gaussian 09, M. J. Frisch, G. W. Trucks, H. B. Schlegel, G. E. Scuseria, M. A. Robb, J. R. Cheeseman, G. Scalmani, V. Barone, G. A. Petersson, H. Nakatsuji, X. Li, M. Caricato, A. Marenich, J. Bloino, B. G. Janesko, R. Gomperts, B. Mennucci, H. P. Hratchian, J. V. Ortiz, A. F. Izmaylov, J. L. Sonnenberg, D. Williams-Young, F. Ding, F. Lipparini, F. Egidi, J. Goings, B. Peng, A. Petrone, T. Henderson, D. Ranasinghe, V. G. Zakrzewski, J. Gao, N. Rega, G. Zheng, W. Liang, M. Hada, M. Ehara, K. Toyota, R. Fukuda, J. Hasegawa, M. Ishida, T. Nakajima, Y. Honda, O. Kitao, H. Nakai, T. Vreven, K. Throssell, J. A. Montgomery, Jr., J. E. Peralta, F. Ogliaro, M. Bearpark, J. J. Heyd, E. Brothers, K. N. Kudin, V. N. Staroverov, T. Keith, R. Kobayashi, J. Normand, K. Raghavachari, A. Rendell, J. C. Burant, S. S. Iyengar, J. Tomasi, M. Cossi, J. M. Millam, M. Klene, C. Adamo, R. Cammi, J. W. Ochterski, R. L. Martin, K. Morokuma, O. Farkas, J. B. Foresman, and D. J. Fox, Gaussian, Inc., Wallingford CT, 2016.

[4] S. Tian, Z. Huang, J. Tan, X. Cui, Y. Xiao, Y. Wan, X. Li, Q. Zhao, S. Li, C.-S. Lee, Manipulating Interfacial Charge-Transfer Absorption of Cocrystal Absorber for Efficient Solar Seawater Desalination and Water Purification, *ACS Energy Lett.* **2020**, *5*, 2698–2705.
